# Aβ and Tau Regulate Microglia Metabolism via Exosomes in Alzheimer’s Disease

**DOI:** 10.3390/biomedicines10081800

**Published:** 2022-07-27

**Authors:** Yuanxin Zhao, Buhan Liu, Jian Wang, Long Xu, Sihang Yu, Jiaying Fu, Xiaoyu Yan, Jing Su

**Affiliations:** Key Laboratory of Pathobiology, Department of Pathophysiology, Ministry of Education, College of Basic Medical Sciences, Jilin University, 126 Xinmin Street, Changchun 130021, China; zhaoyx19@mails.jlu.edu.cn (Y.Z.); liubh20@mails.jlu.edu.cn (B.L.); wjian21@mails.jlu.edu.cn (J.W.); xulong20@mails.jlu.edu.cn (L.X.); yush19@mails.jlu.edu.cn (S.Y.); fujy21@mails.jlu.edu.cn (J.F.)

**Keywords:** Alzheimer’s disease, amyloid-β, tau, microglia

## Abstract

One of the most striking hallmarks shared by various neurodegenerative diseases, including Alzheimer’s disease (AD), is microglia-mediated neuroinflammation. The main pathological features of AD are extracellular amyloid-β (Aβ) plaques and intracellular tau-containing neurofibrillary tangles in the brain. Amyloid-β (Aβ) peptide and tau protein are the primary components of the plaques and tangles. The crosstalk between microglia and neurons helps maintain brain homeostasis, and the metabolic phenotype of microglia determines its polarizing phenotype. There are currently many research and development efforts to provide disease-modifying therapies for AD treatment. The main targets are Aβ and tau, but whether there is a causal relationship between neurodegenerative proteins, including Aβ oligomer and tau oligomer, and regulation of microglia metabolism in neuroinflammation is still controversial. Currently, the accumulation of Aβ and tau by exosomes or other means of propagation is proposed as a regulator in neurological disorders, leading to metabolic disorders of microglia that can play a key role in the regulation of immune cells. In this review, we propose that the accumulation of Aβ oligomer and tau oligomer can propagate to adjacent microglia through exosomes and change the neuroinflammatory microenvironment by microglia metabolic reprogramming. Clarifying the relationship between harmful proteins and microglia metabolism will help people to better understand the mechanism of crosstalk between neurons and microglia, and provide new ideas for the development of AD drugs.

## 1. Introduction

AD is a progressive chronic neurodegenerative disease characterized by cognitive dysfunction that affects the cognitive and emotional behavior of patients, generally 65 years and older [1]. AD is characterized by two major pathological lesions in the brain, amyloid plaques composed mainly of amyloid-β (Aβ) peptides and hyperphosphorylated tau, and neurofibrillary tangles (NFTs) [2]. NFTs are described as one of the leading brain injuries in AD [3] and AD is the most common type of amyloidosis in which amyloid and insoluble neurofibrillary proteins are abnormally deposited in neurons and glia [4]. This may result in the dysfunction of specific brain subregions or neuronal populations, leading to motor or psychiatric disorders with associated behavioral deficits.

Microglia, as the resident immune cells in the brain, dynamically monitor the microenvironment through the movement process of constantly interacting with neurons [5,6]. This crosstalk between microglia and neurons helps maintain brain homeostasis. In the large-scale genome-wide association studies, more than 20 loci associated with AD have been identified, many of which are expressed or only expressed in microglia or myeloid cells [7,8]. Before the formation of plaques in AD, microglia play a role in promoting early synaptic loss and dysfunction [9]. For example, adenosine, the catabolite of ATP, primarily acts on microglia through adenosine A2a receptor (A2AR) and adenosine A3 receptor [10,11]. The combination of adenosine and microglia A2AR promotes neuroprotection by releasing nerve growth factor, but it also induces the expression of prostaglandin E2 and cyclooxygenase-2 and the release of nitric oxide (NO), causing damage to neurons [12]. In clinical studies, the polymorphism of the microglia fractalkine receptor (CX3CR1) has been found. CX3CR1 is associated with the faster progression of disease symptoms and shorter survival time of patients with late-onset AD, which may be caused by the reduction of microglia neuron communication due to the reduction in the signals of CX3CR1 and its ligand chemokine CX3CL1 [13,14]. In addition, fragments of dead neurons are thought to trigger glial cell-mediated neuroinflammation in AD, increasing neuronal death [15]. Microglia can often be reprogrammed by metabolism to change their phenotype and slow the process of neuronal damage [16].

The crosstalk between neurons and microglia may be the key to induce neuroinflammation, as mitochondrial energy metabolism in neurons is crucial for neuronal homeostasis in AD. Recent studies have shown that microglia monitor and protect neuronal function through special purinergic connections, and the activity of neuronal mitochondria is related to the formation of microglia connections [14]. Furthermore, microglia activation-mediated activation of astrocytes can aggravate neuroinflammation and induce neuronal damage in the AD mouse model [17]. Recent evidence suggests that extracellular vesicles (EVs) secreted by neurons and glia including exosomes play a key role in intercellular communication and neuroinflammation by transporting messenger RNA (mRNA), microRNA (miRNA) and proteins [18,19,20]. Similarly, microglia can secrete extracellular vesicles for cell-to-cell communication [21], and the composition of its content depends on the state of the cells and the nature of the priming stimulus [22,23]. This has further deepened the complexity of the cross-talk between microglia and neurons in neurodegenerative diseases. In the case of bidirectional communication between microglia and neurons, microglia phagocytosis and the production of cytokines and free radicals can also be regulated by the neuronal exosomes [24]. In conclusion, the role of microglia in multiple neuronal pathways is crucial in AD.

In summary, the crosstalk between microglia and neurons is a key factor affecting the progression of AD, and metabolic reprogramming of microglia may be an important link in preventing the occurrence and development of AD. In this review, we describe the regulation of microglia metabolism by the accumulation of Aβ oligomers and tau oligomers, which in turn aggravate neuroinflammation and aggravate neuronal damage. Further exploration of the mechanism of the effects of harmful proteins on microglia metabolism may provide new strategies for the development of targeted drugs and the treatment of neurodegenerative diseases.

## 2. Plaques and Tangles Formed by the Accumulation of Aβ Oligomers and Tau Oligomers Play a Key Neuropathogenic Role Leading to Neuronal Damage

**Figure 1:**
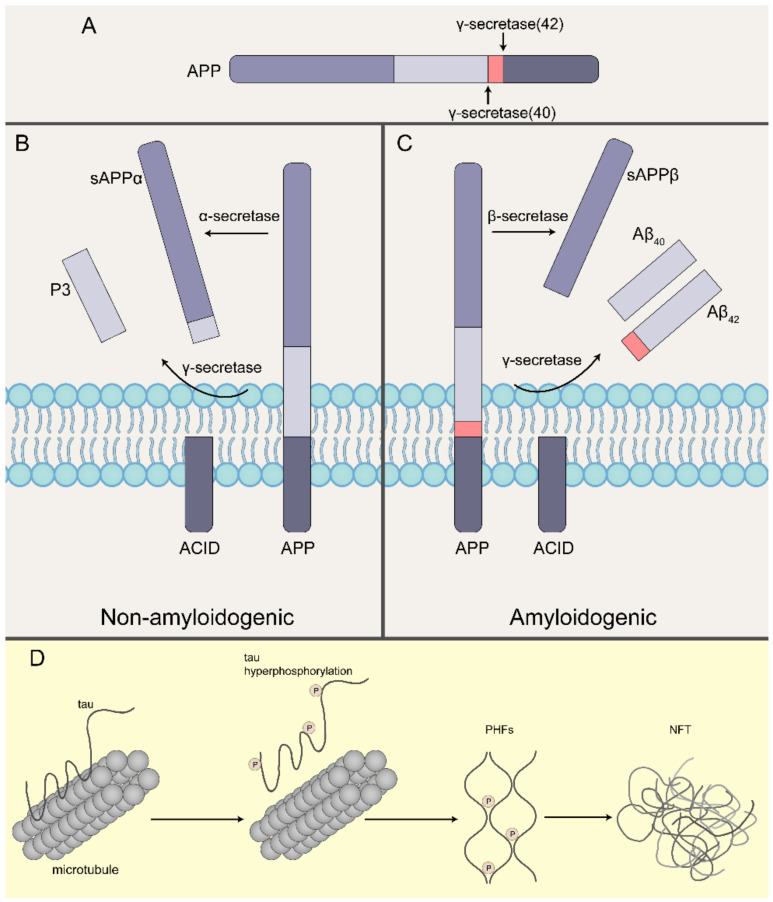
Pathological Aβ and tau formation in Alzheimer’s disease. (**A**) Cleavage sites for γ-secretase. (**B**) In the non-amyloidogenic pathway, APP gets chopped up by α-secretase and γ-secretase, which is called the nonamyloidogenic pathway, resulting in extracellular products called sAPPα and secreted fragment called P3, which are soluble, and leaves the membrane-bound APP intracellular domain (AICD). (**C**) In the amyloidogenic pathway, APP is first cleaved by β-secretase instead followed by the processing by γ-secretase, producing the secreted extracellular products called sAPPβ and Aβ_40_/Aβ_42_, and the same membrane-bound AICD. (**D**) Microtubules are strong cylindrical polymers that provide structural support to neurons. Tau is the major microtubule-associated protein in neurons and it stabilizes microtubule architecture. Under pathological conditions, tau becomes hyperphosphorylated and detaches from microtubules. Phosphorylated tau then aggregates to form paired helical filaments (PHFs) and NFTs.

Although the cause of AD is not fully understood, two major factors that are often cited in its progression are plaques and tangles. Aβ denotes peptides of 36–43 amino acids that are the main component of the amyloid plaques found in the brains of people with Alzheimer’s disease [25]. The imprecise cleavage of γ-secretase at C-terminus of Aβ sequence results in two major Aβ isoforms: Aβ_42_ (42 residues long) and Aβ_40_ (40 residues long). The only difference between them is extra isoleucine and an alanine at the C-terminus of Aβ_42_ [26]. However, the toxicity of Aβ_42_ is much more toxic than Aβ_40_ due to its greater tendency to fibrillise [25,27]. Currently, most people suggested that the mechanism of plaques formation is Aβ accumulated, which was called the “amyloid cascade hypothesis” (Figure 1A,B). Aβ is typically produced by cleavage of amyloid precursor proteins (APP). APP is a type 1 transmembrane protein, which helps the neuron grow and repair itself after an injury [28]. Normally, APP is chopped up by α-secretase and γ-secretase, then formed extracellular soluble products called sAPPα and a secreted fragment called p3, the membrane-bound intracellular domain of APP (AICD). This whole process called the non-amyloidogenic pathway. However, in the amyloidogenic pathway, APP is first cleaved by β-secretase instead of the processing by γ-secretase, producing extracellular products called sAPPβ and Aβ, and the same membrane-bound AICD. The leftover fragment creates a monomer called Aβ, and the monomer tends to be more chemically ‘sticky’. The monomeric Aβ spontaneously assembles into soluble oligomers, and the oligomers are clustered to form insoluble fibrils, which are called β-amyloid plaques [29]. These plaques potentially get between the neurons and make brain functions impaired. In addition, plaques can initiate an immune response and cause inflammation, such as activation of microglia, which could damage surrounding neurons.

Another big part of AD is tangles, which are actually found inside the cell. Neurons are held together by their cytoskeleton, which is partly made up of microtubules. Tau, a normal, unfolded, highly soluble protein, in normal circumstances, tau is a microtubule-associated protein involved in microtubule stabilization [28,30].

Tau is found primarily in axons where it regulates microtubule polymerization and stabilization [31]. The tau protein contains various phosphorylation sites, and under normal conditions, phosphorylation helps to maintain the cytoskeletal structure [32]. Phosphorylation of Tau serine/threonine effectively regulates the binding affinity of Tau to microtubules. However, hyperphosphorylation of tau is known to contribute to the pathology of Alzheimer’s disease, with approximately 45 specific phosphorylation sites identified in the brain of Alzheimer’s disease patients [32,33]. Anormal phosphorylation of the tau protein changes its structure and hyperphosphorylated tau protein becomes dissociated from neuronal microtubules and accumulates in paired helical filaments, and proteolytic processing leads to the formation of tau oligomers, which stimulate the process of aggregation and form NFTs [34].

Some studies suggest that amyloid-β plaques lies upstream of tau oligomers and triggers tau pathology [2,35]. However, there are also studies that have shown that tau can act independently of Aβ, leading to neurodegeneration [36,37,38]. Summarily, tau and Aβ play a role in parallel pathways, leading to AD.

## 3. Metabolic Reprogramming of Microglia to Aggravate the Neuroinflammatory Microenvironment

Microglia as tissue macrophages of the CNS continuously monitors cerebral parenchyma to detect neuronal activities and alteration of homeostatic processes [39,40,41]. Brain metabolism is tightly controlled to maintain accurate neuronal function [42] that also requires metabolic interaction with microglia [43]. Recent studies have shown that microglia might alter neuronal substrate utilization during neuroinflammation [43].

All macrophages, including activated microglia, are functionally heterogeneous and highly plastic. Microglia, as the immunologically active cells of the brain, are the most important immune defense lines of the central nervous system and play an increasingly important role in maintaining normal brain function. Traditionally, microglia are in a resting state (M0 phenotype) under physiological conditions and play an “immune surveillance” role. Under pathological conditions, microglia are rapidly activated, and the activation is accompanied by changes in transcriptional adaptive functions. Neuroinflammatory (M1 polarization) microglia release pro-inflammatory cytokines, chemokines and neurotoxic factors. The neuroprotective (M2 polarization) microglia achieve neuroprotection by promoting tissue repair and regeneration through expressing anti-inflammatory factors [44]. Clinical studies have shown that overactivated M1 phenotype microglia can cause neuronal disability, damage and degeneration; and play an important role in cerebrovascular and neurodegenerative diseases and neurodevelopmental and mental disorders [16,45,46,47]. Microglia secrete proinflammatory factors including Interleukin-1β [26,27,48,49], Interleukin-6 [49,50,51] and TNFα [49,52,53], along with an increased production of ROS [54,55,56], which can cause damage to the surrounding neuronal cells. In AD, M1 phenotype microglia that secrete pro-inflammatory factors maintain the neuroinflammatory microenvironment and continuously aggravate neuronal damage.

**Figure 2:**
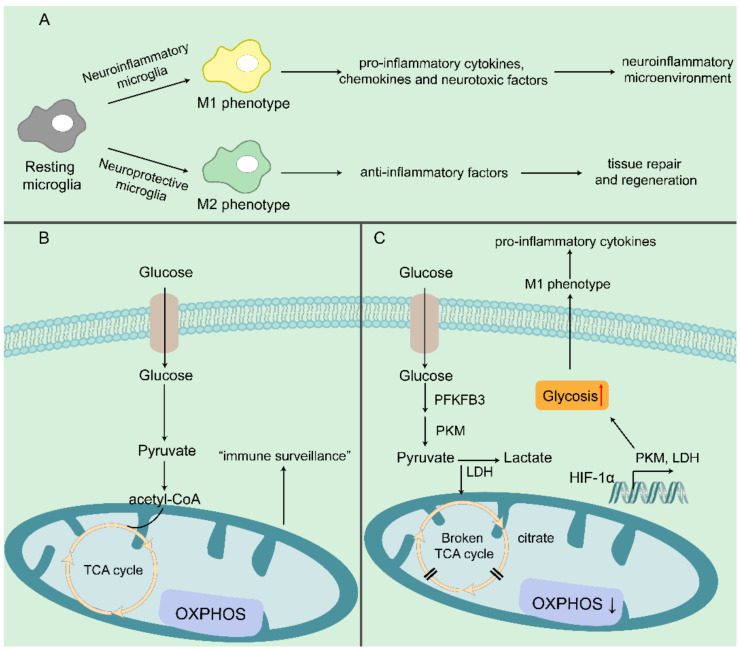
The role of microglia in neuroinflammation. (**A**) In a dichotomy model, the M1-type microglia typically express pro-inflammatory cytokines, chemokines and neurotoxic factors, while the M2-type microglia generally produce anti-inflammatory, neuroprotective and wound-healing factors. (**B**) In the healthy brain, microglia are metabolically flexible and can use glucose to support homeostatic “immune surveillance” functions. (**C**) Under pathological conditions, such as AD, microglia display a metabolic reprogramming featured by broken TCA cycle. HIF1α, hypoxia inducible factor-1α; PKM, Pyruvate kinase isozyme typeM2; PFKFB3, phosphofructokinase-2/fructose-2,6-bisphosphatase 3; OXPHOS, Oxidative phosphorylation; LDH, lactate dehydrogenase. Brown rectangle: glucose transporter; yellow cycle: TCA cycle; purple rectangle: oxidative phosphorylation; orange rectangle: increased glycolysis.

The metabolic phenotype determines the polarizing phenotype. Microglia undergo comprehensive phenotypic remodeling to adapt effector functions [43]. To perform this multitude of functions, microglial metabolism must be precisely regulated to ensure adequate energy supply and synthesis of response-specific molecules [57]. Therefore, microglia are metabolically flexible to reprogram the metabolism of microglia to change the neuroinflammatory microenvironment and block the aggravation of neuronal damage. M1-type pro-inflammatory microglia cells showed increased glycolytic metabolism and impaired OXPHOS, while M2-type anti-inflammatory macrophages showed increased OXPHOS (Figure 2). Model M1 utilizes this anabolic metabolism to produce much lower ATP than OXPHOS, necessary for balancing energy production with macromolecular synthesis [45,58,59]. In addition, glycolysis provides food for the pentose-phosphate pathway (PPP). PPP supports the production of amino acids for protein synthesis, ribose for nucleotides, and NADPH needed to produce ROS. In M1 microglia, TCA is interrupted, and the intermediate (acetyl-CoA) induces fatty acids, lipids, and prostaglandin synthesis or is used to induce cytokine production [60]. In the proteomic study on AD of Johnson et al. [61], the module of the protein network linked to glycometabolism emerged as one of the most significantly associated modules with AD pathology and cognitive impairment. Also, the enrichment of microglial proteins in the glucose metabolism module were observed. Among them, glycolysis-related proteins were increased, including lactate dehydrogenase (LDH), pyruvate kinase M(PKM) and glyceraldehyde 3- phosphate dehydrogenase (GAPDH). In addition, Abhishek JHA et al. found that the interruption of TCA cycle resulted in the accumulation of citrate and succinate by coordinating the integration of metabolomics and transcription, and revealed that glutamine metabolism was characterized by M2 polarization [62]. Up-regulation of iNOS and NO production in M1-type macrophages, which nitrosylates proteins in the mitochondrial electron transport chain, leads to suppression of OXPHOS [57]. In most cases, however, the blood-brain barrier prevents the influx of alternative substrates (fatty acids, amino acids, ketones) and therefore the brain needs a continuous supply of glucose. As blood glucose levels decrease, ketones produced by peripheral fatty acid metabolism supplement glucose as an energy source [46]. Fatty acid oxidation (FAO) fuels OXPHOS, and has been shown to affect polarization [63]. Carroll et al. [64] demonstrated in bone marrow-derived macrophages that CoA promotes the localization of TLR4in the lipid raft cell membrane, linking fatty acid synthase to pro-inflammatory macrophage activation.

Polyamine metabolism of microglia can also regulate its energy metabolism. In AD patients, there is typical up-regulation of arginase (ARG), arginine brain deprivation, and a large increase in brain polyamine levels. Arginine generates ornithine by the action of arginase; ornithine generates putrescine by the action of ornithine decarboxylase, and spermidine is generated by spermidine synthase. One of the functions of spermidine is to participate in the post-translational modification of eIF5A to form hydroxybutylamine-modified hypusine, abbreviated as eIF5A^H^. This modification is necessary for the function of eIF5A. Therefore, the M2-type polarization of macrophages dependent on oxidative phosphorylation is eIF5A^H^-dependent, which in turn enhances the arginase activity, increases the arginine-converted spermidine, and further enhances its oxidative phosphorylation, and maintains its M2 phenotype. Studies have shown that polyamine metabolism and arginine metabolism of microglia are dependent on the change of polarization phenotype [65,66,67,68,69].

In summary, the harmful proteins tau and Aβ can directly regulate the energy metabolism of microglia, and indirectly affect its energy metabolism through FAO, glutamine metabolism, and polyamine pathway. The polarization of microglia depends on the metabolic pattern of mitochondria, which regulates the polarization of microglia. Therefore, tau and Aβ regulating microglia energy metabolism, and neuron–microglia crosstalk may be new therapeutic approaches for AD.

## 4. Aβ and Tau as a Regulator of Microglia Metabolism Reprogramming

The relationship between tau and Aβ and microglia metabolism has been confirmed at this stage (Table 1). Microglia can recognize Aβ oligomer and tau as the danger signal. Sung et al. cultured pure primary microglia from neonatal mouse brain tissues. The PMGs were then treated with various forms of Aβ, including monomeric Aβ, oligomeric Aβ and fibrillary Aβ, all forms of Aβ altered the morphology of the microglia, Aβ substantially increased the expression of pro-inflammatory cytokines protein and mRNA [70]. Oligomeric tau was co-located with microglia in animal models as well as in the brains of AD patients using immunofluorescence by Ashley N. Nilson et al. [71]. What is more, people believed Aβ and tau could induce mitochondrial toxicity and metabolic dysfunction. Aβ induces metabolic reprogramming of microglia from OXPHOS to glycolysis, which is dependent on the mTOR-HIF-1α pathway [70]. The mTOR pathway regulates glucose metabolism as part of a mechanism for sensing the energy status of the cell. Phosphorylated mTOR increases the expression of HIF-1α, the master transcriptional regulator of glycolysis [72]. Rosella Abeti et al. found that Aβ affects mitochondrial oxygen consumption and that cells appear to deplete NADH [73]. Joshi AU et al. found iron accumulation in microglia isolated from APP/PS1 mice and in primary IFN+Aβ-treated mouse microglia, with decreased ability to engulf Aβ with increased glycolysis [74]. Consistent with microglia effects, studies have shown that IFNγ+Aβ increases the expression of HK II and PKM2, which catalyze the first and last reactions in glycolysis, while IFNγ+Aβ also increases PFKFB3. PFKFB3 catalyzes the production of fructose 2,6-diphosphate, which is the allosteric activator of phosphofructokinase. PFKFB3 is recognized as a key glycolysis regulator, responsible for the conversion of fructose-6-phosphate to fructose-1,6-diphosphate, a rate-limiting irreversible glycolysis [75,76].

Receptors on microglia that recognize Aβ and tau can also participate in the regulation of metabolic reprogramming. Trigger receptor 2 (TREM2) expressed on myeloid cells is a microglia receptor that recognizes changes in the lipid microenvironment that may occur during Aβ accumulation and neuronal degeneration of AD. The risk of AD is related to TREM2 receptor subtype variants on microglia. Yingjun Zhao et al. proved that the extracellular domain of TREM2 can bind to Aβ [111]. Using genome-wide RNA sequencing and multiphoton microscopy, we further identified metabolically deficient microglia in 5XFAD mice and found that exposure to Aβ triggered acute microglia inflammation with metabolic reprogramming from OXPHOS to glycolysis, following the discovery by Wang Y et al. in a 5XFAD mouse, an AD mouse model, that Aβ affected microglia activation through the receptor TREM2 [70,82]. The study of Tyler K. Ulland et al. showed that TREM2 deficiency impairs mTOR signaling and enhances AMPK activation in microglia. Furthermore, gene expression microarray analyses of sorted microglia from Trem2 KO 5XFAD and 5XFAD mice revealed that TREM2 deficiency was associated with decreased expression of genes encoding translation initiation factors, ribosomal proteins, glucose transporters, glycolytic enzymes, as well as the transcription factor HIF1α that controls glycolysis [77]. TREM2 can also over-activate AKT [78], and then promote glycolysis through mTOR/HIF1α pathway [70]. Similarly, tau can also affect the metabolism of microglia through TREM2 [93,94,95].

The Toll-like receptor can recognize Aβ and activate microglia [83], and promote metabolic reprogramming by activating the ATP-Citric Acid Lyase (Acly)-driven glucose uptake and cytosolic synthesis of acetyl coenzyme A and oxaloacetic acid [84], or up-regulating PFKFB3 expression [76].Consistent with this, an analysis of resting brain PET by S. Neil Vaishnavi et al. [112] and studies of FDG-PET (for quantitative measurement of local human brain glucose metabolism) in AD patients by Andrei G. Vlassenko et al. [113] showed an increased dependence on aerobic glycolysis in regions associated with Aβ deposition space in the brain of AD patients. Cytochrome c oxidase (COX) and adenine nucleotide transporter (ANT) are targets of chemically synthesized pathological forms of tau, but ANT is a unique mitochondrial target responsible for OXPHOS damage, discovered by Atlante A et al. [114]; Aβ can also enter the mitochondria through the outer membrane transposase (TOM) [115] and cyclophilin D [116] which is a component of the mitochondrial permeability transition pore, and then binds to alcohol dehydrogenase [117], thus increasing Ca^2+^ influx [117] and TCA cycle disorders (such as abnormal metabolism of α -ketoglutarate [118], affecting the metabolism of microglia [119]. Aβ can also interact with formyl peptide receptors on microglia, and the activation of formyl peptide receptors can then activate the AMPK pathway and inhibit the activation of the mTOR pathway to regulate the metabolism of microglia [79]. The RAGE receptor is related to the recognition of Aβ by microglia. Rashid Deane et al. found that when RAGE receptor was blocked, the pro-inflammatory factors secreted by microglia induced by Aβ decreased [120]. Tau activation of microglia is related to apolipoprotein e gene (APOE) [96,97,98]. APOE may affect many metabolic pathways, such as lipid metabolism [98,100], glucose metabolism [100,101].

Aβ-mediated epigenetic modifications may also be involved in the regulation of microglial metabolism. Lysine acetylation regulates key metabolic enzymes, which thereafter regulate immune processes. Histone deacetylases (HDACs) can act as transcriptional repressors of genes, which regulate glycolytic enzymes and their activity has been linked to inflammatory responses but it is not clear [121]. SITR1 is NAD^+^ dependent HDACs. Cell level of NAD^+^ is an important indicator of cell energy state [90]. Grazia Ilaria Caruso et al. found that Aβ can up-regulate the expression of SRIT1 in the nucleus of human microglia cell line HMC3 and activate the enzyme activity of SIRT1 [89]. Lactate is not only a metabolic waste; glycolysis-derived lactate was identified as a substrate for histone lactonization [122] and Rui-Yuan Pan et al. found increased histone lactonization in microglia adjacent to 5XFAD mouse plaques. Moreover, histone lactonization directly stimulated the gene transcription level, and the mRNA level expression of glycolysis-related genes Hif-1α, Pkm2 and Ldha were significantly increased at the same time [91].

In addition, Aβ and tau can indirectly affect metabolism by up-regulating cytokines and pro-inflammatory reactions related to the M1 phenotype through other related signaling pathways, such as IL-1β and IFNγ. Inflammatory cytokine-induced glycolysis program with disruption of the TCA cycle and uncoupling of OXPHOS [80,81,82]. Luxi Wang et al. found that microglia secrete pro-inflammatory cytokines in an inflammatory environment, and thus the expression of GLUT promotes a glucose increase [123]. Furthermore, Casimir Bamberger et al. revealed through Covalent Protein Painting that Aβ was related to inhibition of mitochondrial succinate dehydrogenase, which could be related to racemization of its serine residues [124,125] or to the lysine site of tau [114], which was the basis for the interruption of the TCA cycle in pro-inflammatory microglia, leading to the accumulation of succinic acid [126]. Meihua Jin et al. demonstrated that tau could activate the cGAS/STING pathway in microglia [104], while STING activation could lead to the accumulation of the macrophage metabolite succinate. This stabilizes the transcription factor HIF-1α, leading to a shift to the glycolytic profile [105]. Mammalian targets that inhibit rapamycin complex 1 (mTORC1) can inhibit hexokinase 1-dependent glycolysis and caspase-1 activation, which indicates that inflammasome activation of NLRP3 is involved in macrophage metabolism [127]. Aβ [85,86,87,88] and tau [107,108,109,110] can activate NLRP3 of microglia via converting its metabolism into sugar [107,127]. Bradley L. Heckmann et al. activated RAW264.7 and BMDM at Aβ is closely related to TREM2 and CD63 [128]. CD36 is a type B scavenger receptor that recognizes low density lipoprotein (LDL), oxidized phospholipid and Aβ, and is also considered a FA transporter or uptake promoter [43]. These findings suggest that AD-related stimuli can directly change the metabolic processes of microglia in a variety of ways. However, the precise signaling mechanism involved remains to be fully elucidated.

## 5. Exosomes as the Mediator of Aβ and Tau Propagation

Neuronal aggregates of tau and Aβ can generally act on adjacent microglia through exosomes or receptors. Exosomes are membrane-bound vesicles released by mammalian cells from the intracellular multivesicular compartments. They play an important role in intercellular communication because exosomes are potential vehicles for transferring cell contents from source cells to recipient cells [129]. In AD, exosomes are related to the release of Aβ oligomer and its extracellular accumulation [130,131,132,133], and the accumulation of hyperphosphorylated tau [134,135,136,137]. In neuronal cytoplasm, APP is endocytosed and cleaved by β-secretase to form APPβ on the early endosome membrane. This early endosome undergoes maturation to form multivesicular bodies (MVB)-containing exosomes, formed by the invagination of early endosome during maturation. Tau protein oligomers are firstly passed through endoplasmic reticulum and Golgi body, and then they become part of secretary vesicles and later exosomes. Under physiological conditions, these Tau- and Aβ-containing exosomes are degraded by lysosomes. But in the case of AD, when there is a problem in lysosomes, then these MVBs containing exosomes are filled with Tau and Aβ, and are released into the extracellular space. From the extracellular space, exosomes are captured by microglia [138].

Many studies have proved that neuron-derived exosomes contain Aβ oligomers and tau, and can be internalized by adjacent cells, activating the immune response or propagation (Table 2). Maitrayee et al. detected exosomes containing Aβ oligomers in the brain of human AD patients and demonstrated that t exosomes could be a vehicle for the spreading of Aβ oligomers [137]. Consistent with this, Aβ and tau were detected in plasma neuron-derived exosomes [133] and exosomes in the cerebrospinal fluid of AD patients [139]. In the AD mouse model, Ahmed et al. isolated Aβ-related exosomes and found that they could be transported to mitochondria [140]. Rajendran et al. demonstrated in neuroblastoma N2a cells that a fraction of Aβ peptides is localized to MVBs and is released in association with exosomes, and Aβ was still seen on the exosomes from the cryoelectron microscopy specimen after several stringent washing which demonstrates that it is not that extracellular soluble Aβ simply ‘‘sticks’’ to the extracellular exosomes, but that Aβ is contained in the exosomes or that Aβ is inserted in the membrane [141]. Saman S et al. detected the presence of tau in isolated exosomes from cerebrospinal fluid of human AD patients and verified in M1C cells (neuroblastoma) that most of their secreted tau occurred through exosome release [142]. Similarly, Fiandaca MS et al. detected tau and Aβ in neurogenic blood exosomes extracts from AD patients [143]. Guix FX et al. contained aggregates of tau in the exosomes of neurons derived from human induced pluripotent stem cells (iPSC) and detected several different forms of tau in plasma-isolated exosomes from AD patients [144]. However, the mechanism by which Aβ and tau are encapsulated in exosomes is not yet clear.

In addition, Aβ and tau are not only found in exosomes, but also internalized by adjacent cells. Charisse Winston et al. confirmed whether exosomes spread the pathological forms of tau in vivo [144]. Wang Y et al. found that exosomes containing tau in the conditioned medium from organotypic hippocampal slices seeded tau accumulation in adjacent microglia [148]. Microglia depletion was found to inhibit the propagation of tau in a P301S tau transgenic mouse model by Asai H et al. [149]. The synergy of Aβ recognition, internalization and clearance, and cell activation may involve microglia receptors, such as scavenger receptors (SR-AI/II), CD36, RAGE, Fc receptors, TLR (Toll-like receptor), and complement receptors. Exosomes boosted the uptake of Aβ by primary cultured microglia in vitro, discovered by Michael B Dinkins et al. in AD 5XFAD mouse model [152]. Crehan et al. treated primary rat microglia with Aβ after blocking complement receptor 1 and found reduced microglia activation [153]. Ahmed Elsherbini et al. found in the AD 5XFAD mouse model that exosome-mediated Aβ acted on mitochondria, binding to and aggregation of mitochondrial outer membrane protein 1(VDAC1) [140]. Indra Sethy Coraci et al. verified the expression of CD36 in microglia in the brain of AD patients and confirmed the binding of Aβ to CD36 in the Bowes-CD36 cell line, which was transfected with human CD36 mammalian expression vector and produced a cell line expressing CD36 [154]. Microglia surface receptors are involved in the internalization of Aβ and tau in exosomes, consistent with their involvement in reprogramming of microglia metabolism.

## 6. Conclusions

**Figure 3:**
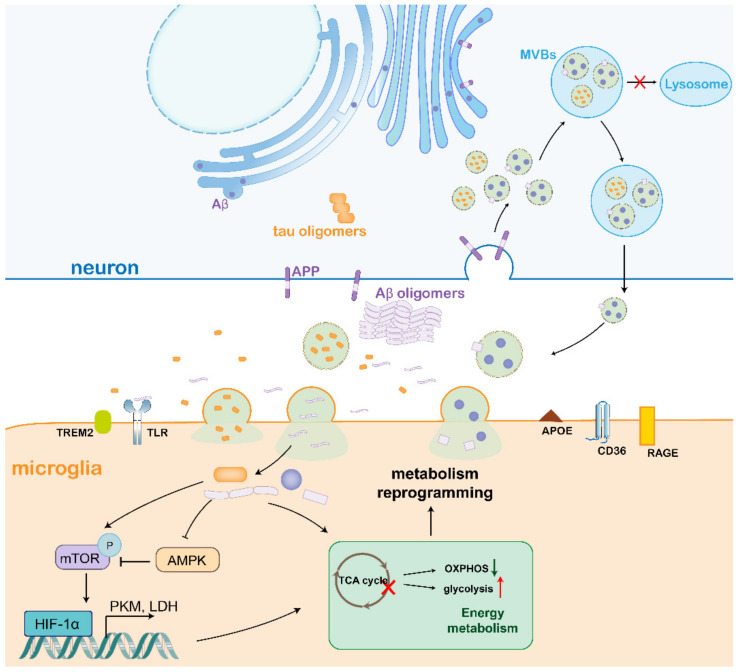
Tau and Aβ proteins, which are, accumulated inside the cytoplasm of the neuron gets encapsulated inside the exosomes in MVBs. These Tau- and Aβ-containing exosomes are degraded by lysosomes, but in AD when there is a problem in lysosomes; then these MVBs containing exosomes are filled with Tau and Aβ, and are released into the extracellular space. From the extracellular space, exosomes are captured by microglia. In addition, the accumulated Aβ oligomer and tau oligomer may act on microglia adjacent to neurons with free forms, and cause metabolic reprogramming of microglia through receptors or related signal pathways, thus maintaining the neuroinflammatory microenvironment. Purple cycle: intracellular Aβ; purple rectangle: APP; light purple wavy rectangle: Aβ oligomers; orange rectangle: tau; green rectangle: TREM2; brown triangle: APOE; yellow rectangle: RAGE.

In conclusion, the inflammatory microenvironment mediated by microglia is crucial for the onset and progression of AD. The aggregates, in combination with Aβ and tau accumulation, can cause neuronal dysfunction and glial activation, followed by neuroinflammation in AD. Aβ and Tau are produced in neurons and released into the extracellular space, where they can be degraded or cleared by microglia, or enter microglia via receptors on their surface to promote glycolysis by regulating mitochondrial function, aggravating the neuroinflammatory microenvironment and neuron injury. Therefore, it has previously been observed that it is urgent to explore the means for the treatment of neurodegenerative diseases by reprogramming microglia metabolism (Figure 3).

The regulation of Aβ and tau on microglia metabolism is mainly focused on the effect on mitochondrial function. Mitochondrial metabolism, which includes the tricarboxylic acid cycle, OXPHOS, FAO, nucleotide synthesis, and amino acid metabolism, is at the core of the cellular metabolic network. Both Aβ and tau mutations can cause mitochondrial dysfunction, and Aβ has been identified as a crucial role in the formation of free radicals, oxidative damage, and mitochondrial dysfunction [155]. The development of Aβ plaques has been proven to cause severe damage to mitochondria in studies. The ETC function of mitochondria can be exacerbated by the combined impacts of Aβ and ROS, a break-down of the electron transfer cycle by preventing cytochrome oxidase from producing reactive oxygen species (ROS) [156,157,158]. Lener et al. utilized the respiratory inhibitors rotenone and antimycin to create mitochondrial damage and increase the quantity of ROS in neural cells, and discovered that the level of A was also rising. In the brain of AD patients, the activities of various mitochondrial localization enzymes decreased, and in most neurons, the number of intact mitochondria decreased [159]. The changes in some mitochondrial enzyme activities found in AD are related to the pathology of Aβ [160]. Recent studies have found that Tau oligomers alter memory consolidation, reduce the levels of synaptophysin and neuron-specific protein 11 associated with synaptic vesicles, and also reduce the level of Complex I. Brain samples from pR5 transgenic mice overexpressing tau protein mutant P301 showed that mitochondrial complex enzyme activity decreased, mitochondrial depolarization, respiratory damage and ROS level increased [161]. It is suggested that Tau protein or Tau fragment can directly or indirectly affect the activity or expression level of mitochondrial respiratory enzymes, and make mitochondrial function disorder.

In addition, in the inflammatory environment in which neurons are located, not only the microglia play a role, but also the crosstalk between microglia and astrocytes plays an important role. Microglia generally respond faster than astrocytes to pathological stimuli, inducing astrocyte activation and determining the fate of astrocytes [162]. Microglia under the condition of neuritis release ATP and stimulate astrocytes to release glutamic acid. Glutamic acid is a well-recognized modulator of neuronal excitability, which can act, for example, through metabolic glutamate receptors, indicating that microglia play an important role in the regulation of astrocyte-mediated excitatory neurotransmission [163]. Activated microglia negatively affected the activity and expression of the glutamic acid-aspartic acid transporter in cultured astrocytes [164]. On the other hand, inflammatory mediators released by activated microglia increase the uptake of extracellular glutamate, which is dependent on glutamate transporter 1, in astrocytes [165]. Increased extracellular glutamate uptake in the inflammatory response is associated with increased expression of GLUT1. Aβ and tau interact strictly with microglia and neurons in the microenvironment of neuroinflammation, and microglia metabolism reprogramming is the central point for regulating neuroinflammation. Reversing microglia phenotype by targeting microglia metabolism may provide a new strategy for the treatment of AD.

## Figures and Tables

**Table 1:** Effect of Aβ oligomers and tau oligomers on microglia metabolism.

	Receptor	Signaling Pathway	Metabolism	References
Aβ		mTOR/HIF1α	up-regulation of glycolysis-related protein transcription	[70]
	TREM2	Akt/mTOR/HIF1α	up-regulation of glycolysis-related protein transcription	[77,78]
	Formyl peptide receptor	AMPK/mTOR	up-regulation of glycolysis-related protein transcription S	[79]
	RAGE Receptor	Inflammatory cytokines	Interruption of TCA cycle and uncoupling of OXPHOS	[80,81,82]
	Toll-like receptor		Up-regulation of the expression of PFKFB3; activation of ATP- citrate lyase; down-regulation of Arg1	[76,83,84]
		NLRP3	Increased glycolysis	[76,85,86,87,88]
		SRIT1	Increased glycolysis	[89,90]
		Histone lactylation	Up-regulation of transcriptional levels of glycolysis related genes HIF1α, PKM2 and LDHA	[77,91]
	CD36		FA transporter or uptake promoter	[43,92]
Tau	TREM2	Akt/mTOR/HIF1α	up-regulation of glycolysis-related protein transcription	[93,94,95]
	APOE		Lipid metabolism; glucose metabolism	[96,97,98,99,100,101]
		p38 AMPK	Up-regulation of the expression of PFKFB3	[102,103]
		cGAS/STING	accumulation of the metabolite succinate	[104,105]
		NLRP3	Increased glycolysis	[106,107,108,109,110]

Abbreviations: mTOR, mammalian target of rapamycin; HIF1α, hypoxia inducible factor-1α; TREM2, Triggering Receptor Expressed On Myeloid Cells 2; Akt, Serine/Threonine Kinase; AMPK, Adenosine 5‘-monophosphate (AMP)-activated protein kinase; RAGE, the Receptor of Advanced Glycation EndproductsTCA, tricarboxylic acid; OXPHOS, Oxidative phosphorylation; PFKFB3, phosphofructokinase-2/fructose-2,6-bisphosphatase 3; Arg1, Arginase 1; NLRP3, NOD-like receptor thermal protein domain associated protein 3; SRIT1, Sirtuin 1; PKM2, Pyruvate kinase isozyme typeM2; LDHA, lactate dehydrogenase A; APOE, Apolipoprotein E; FA, fatty acid; cGAS, Cyclic GMP-AMP Synthase; STING, Stimulator Of Interferon Response cGAMP Interactor.

**Table 2:** AD-associated exosomes contain Aβ and tau.

Protein	Location	Species	The Uptake of Adjacent Cells	References
Aβ	SH-SY5Y cells and rat primary neurons	APP, AICD, C-terminal fragments (CTF)		[145]
	N2a cells	Aβ		[141]
	CHO-APP_695_ cells	CTF, APP, Aβ		[146]
	APP transgenic mice, brain from AD patients	CTF, APP, Aβ		[147]
	brain samples of temporal neocortex from AD subjects, SH-SY5Y cell	Aβ oligomers	Peripheral neurons	[137]
Tau	M1C cells and CSF samples from patients with AD	Phosphotau Species Associated with Neurodegeneration/dimerized or trimerized tau species		[142]
	N2a cells, rat primary neurons and CSF of patients with AD	Hypophosphorylated tau	Peripheral neurons	[148]
	transgenic mice with rapid tau propagation		Peripheral microglia	[149]
	lamprey CNS	hyperphosphorylated tau		[150]
	PS19 mice, CSF of patients with AD	p-Tau	Peripheral microglia	[151]
Aβ and tau	plasma or serum from AD patients	P-S396-tau, P-T181-tau, and Aβ1-42		[133,143]
